# Evaluation of a Tap-Based Smartphone App for Heart Rate Assessment During Asphyxia in a Porcine Model of Neonatal Resuscitation

**DOI:** 10.3389/fped.2019.00453

**Published:** 2019-11-05

**Authors:** Peter A. Johnson, Nicolò Morina, Megan O'Reilly, Tze-Fun Lee, Po-Yin Cheung, Georg M. Schmölzer

**Affiliations:** ^1^Centre for the Studies of Asphyxia and Resuscitation, Neonatal Research Unit, Royal Alexandra Hospital, Edmonton, AB, Canada; ^2^Department of Pediatrics, Faculty of Medicine and Dentistry, University of Alberta, Edmonton, AB, Canada

**Keywords:** infants, newborn, neonatal resuscitation, heart rate, smartphone App, auscultation

## Abstract

**Objectives:** Heart rate (HR) is the most significant parameter to assess a newborn's clinical status at birth. Recently, novel technologies including smartphone applications have been suggested for HR assessment during neonatal resuscitation. The aim of this study was to evaluate the accuracy, speed, and precision of the NeoTapLifeSupport (NeoTapLS) smartphone application using a digital stethoscope (DS) for HR assessment during neonatal resuscitation.

**Design:** Newborn piglets (*n* = 20, 1–3 days, 1.7–2.4 kg) were anesthetized, intubated, mechanically ventilated, and subjected to 30 min of hypoxia, followed by asphyxia. Asphyxia was induced by clamping the endotracheal tube and disconnecting the ventilator, until asystole was confirmed by zero carotid blood flow (CBF).

**Setting:** Experimental setting.

**Subjects:** Asphyxia-induced newborn piglets.

**Interventions:** During asphyxia, HR assessments were performed with a DS using the NeoTapLS smartphone application, and compared to 6-s method (6 s), and 10-s method (10 s).

**Measurements and Main Results:** Accuracy of obtained HRs was compared to CBF and electrocardiogram and assessment time using NeoTapLS, 6 s, and 10 s were also measured. The mean(SD) HR with the NeoTapLS was 68(26), compared to CBF with 68(27) bpm, 6 s with 68(27), and 10 s with 66(26) bpm during asphyxia. Bland-Altman analysis revealed no difference between HR using the NeoTapLS, 6 s, 10 s, compared to CBF HR, with NeoTapLS showing the smallest difference between 95% limits of agreement. The median (IQR) time required to obtain a HR using the NeoTapLS was 3(2–4) s, compared to 6(6–7), and 10(10–11) s for 6 and 10 s, respectively.

**Conclusions:** Our data suggests that the NeoTapLS is accurate, fast, and precise during neonatal asphyxia to assess heart rate.

## Introduction

The fetal-to-neonatal transition is a significant challenge at birth, which depends on effective lung aeration and onset of breathing (1, 2). The transition is a sequence of physiological events where liquid must be cleared from the lung, hemodynamic changes such as increase in pulmonary blood flow and systemic vascular resistance, occlusion of fetal shunts, and increases in heart rate (HR) occur (3–5). Asphyxia at birth is the most common reason that newborn infants fail to make a successful transition, as it can depress myocardial function and act against this sequence inducing bradycardia, which leads to asystole (cardiac arrest) (6). HR is therefore the most important parameter to assess a newborn infant's clinical status at birth. Assessment of HR is used to determine the timing, type and efficacy of respiratory support interventions that are needed during neonatal resuscitation (7, 8). It is imperative that HR be continuously monitored in newborns at risk for asphyxia, both rapidly and accurately. If HR detection is slow or overestimated, an intervention might be delayed or prolonged; alternatively, if HR is underestimated, it might result in inappropriate interventions.

The Neonatal Resuscitation Program (NRP) and European Resuscitation Council (ERC) Guidelines for Resuscitation recommends the combined use of pulse oximetry and electrocardiogram (ECG) for HR assessments throughout resuscitation (7–11). Pulse oximetry and ECG are routinely used to continuously monitor HR during neonatal resuscitation. However, on average it takes ~90–120 s and 30–100 s to setup, detect and acquire a reliable HR signal after delivery using pulse oximetry and ECG, respectively (12–17). This is concerning because the first minutes of life are most critical for a newborn requiring assistance and when HR must be assessed quickly to decide what interventions are needed. Additionally, ECG and pulse oximetry are costly and might be inaccessible in resource-limited regions of the world. The NRP additionally recommends combining palpation and auscultation for HR assessment with pulse oximetry and ECG, while the ERC only suggests the use of palpation for assessing signs of recovery during resuscitation (9–11). While palpation and auscultation using a stethoscope can assess HR faster compared to ECG and pulse oximetry, these techniques notably underestimate HR by 8 and 13%, respectively, when compared to ECG (18).

More recently, cost-effective and universally accessible assistive technology (e.g., tap-based smartphone and mobile apps) represent another approach to assess HR in newborn infants (16). Simulation studies reported that it is feasible to assess HR using tap-based smartphone applications (19–21). However, this technology has yet to be tested *in vivo* or during asphyxia in a neonatal model. The aim of this study was to assess the accuracy, speed, and precision of NeoTapLifeSupport (NeoTapLS), a tap-based smartphone application for HR assessment during asphyxia in our porcine model of neonatal resuscitation for the first time. We hypothesized that NeoTapLS would have a similar accuracy compared to HR assessed by carotid blood flow (CBF).

## Materials and Methods

Twenty newborn mixed breed piglets (1–3 days of age, weighing 2.0 ± 0.4 kg) were obtained on the day of experimentation from the University Swine Research Technology Center. All experiments were conducted in accordance with the guidelines and approval of the Animal Care and Use Committee (Health Sciences), University of Alberta (AUP2151), presented according to the ARRIVE guidelines (22), and registered at preclincialtrials.eu (PCTE155).

### Animal Preparation

Piglets were instrumented as previously described with modifications (23–26). Following the induction of anesthesia using isoflurane, piglets were intubated via a tracheostomy, and pressure-controlled ventilation (Acutronic Fabian HFO; Hirzel, Switzerland) was commenced at a respiratory rate of 16–20 breaths/min and pressure of 20/5 cmH_2_O. Oxygen saturation was kept within 90–100%, glucose level and hydration was maintained with an intravenous infusion of 5% dextrose at 10 mL/kg/h. During the experiment anesthesia was maintained with intravenous propofol 5–10 mg/kg/h and morphine 0.1 mg/kg/h. Additional doses of propofol (1–2 mg/kg) and morphine (0.05–0.1 mg/kg) were given as needed. The piglet's body temperature was maintained at 38.5–39.5°C using an overhead warmer and a heating pad.

### Haemodynamic Parameters

A 5-French Argyle^®^ (Klein-Baker Medical Inc. San Antonio, TX) double-lumen catheter was inserted via the right femoral vein for administration of fluids and medications. A 5-French Argyle^®^ single-lumen catheter was inserted above the right renal artery via the femoral artery for continuous arterial blood pressure monitoring in addition to arterial blood gas measurements. The right common carotid artery was also exposed and encircled with a real-time ultrasonic flow probe (2 mm; Transonic Systems Inc., Ithica, NY) to measure CBF. Piglets were placed in supine position and allowed to recover from surgical instrumentation until baseline haemodynamic measures were stable (minimum of 1 h). Ventilator rate was adjusted to keep the partial arterial CO_2_ between 35 and 45 torr as determined by periodic arterial blood gas analysis. Mean systemic arterial pressure, systemic systolic arterial pressure, HR, and percutaneous oxygen saturation were continuously measured and recorded throughout the experiment with a Hewlett Packard 78833B monitor (Hewlett Packard Co., Palo Alto, CA) and LabChart^®^ programming software (ADInstruments, Houston, TX, United States).

### ECG and CBF

A 3-lead ECG was placed on skin at the right fore limb, left fore limb and left hind limb. In addition to its use for HR monitoring, CBF can also be utilized to calculate HR. While ECG HR is commonly used as a clinical gold standard, CBF HR was selected as the experimental gold standard, which offers a comparatively better measure of HR in the setting of asphyxia and in the likely event of pulseless electrical activity (27). However, both ECG and CBF were used as standard for HR comparisons.

### Digital Stethoscope

The digital stethoscope (DS) is a form of electronic stethoscope, which functions by converting the audial heartbeat signal into an electronic signal followed by amplification to provide clearer detection (16, 28). Several studies report the DS is not affected by the use of respiratory support and can be utilized for HR assessment in newborn infants with better accuracy, compared to a standard stethoscope (16, 29, 30).

Auscultation was performed using a DS (Thinklabs One, Denver, CO). Assessments were performed using both the (i) 6-s method (6 s) and ii) 10-s method (10 s). The 6 s method is currently recommended by the NRP (11), whereby HR is calculated by multiplying the number of heartbeats heard in 6 s by 10. The 10 s method has previously been recommended for initial HR assessment at birth (31, 32), and calculates HR by multiplying the number of heartbeats heard in 10 s by 6. The frequency filter of the DS was set between 30 and 500 Hz, which produces low frequency heart sounds and filters out lung sounds, and amplification was set to 6 on the 0–10 Scale.

### Tap-Based Smartphone App

The NeoTapLifeSupport (NeoTapLS; Tap4Life, Stockholm, Sweden) smartphone app was downloaded from App Store (Apple, Cupertino, CA) and paired with the digital stethoscope for HR assessments. NeoTapLS is a recent development for HR assessment, however has only been tested in high-fidelity resuscitation simulation scenarios (16, 19–21). NeoTapLS displays a HR generated by at least three taps on the smartphone screen, which coincides with what the healthcare provider auscultates. Based on this predefined calculation algorithm, when HR is 30 bpm, a minimum of 6 s is required to assess HR [3^*^(60/30) = 6 s], and at 18 bpm, it will take a minimum of 10 s [3^*^(60/18) = 10 s]. Thus, it was expected to be faster than the 6 and 10 s method on average. The time needed to display HR from the first tap until the app assessed HR was recorded.

### Experimental Protocol

Following at least 1 h of stabilization after the surgical protocol, piglets were subjected to 30 min of nitrogen-induced hypoxia (FiO_2_ 10–15%). Hypoxia was then followed by asphyxia until asystole, achieved by disconnecting the ventilator and clamping the endotracheal tube. Asystole was defined as no audible HR during auscultation for at least 10 s and zero CBF. All HR assessments were performed during the asphyxia time leading to asystole (i.e., between disconnecting the ventilator and clamping the endotracheal tube and confirmation of asystole) and were performed by a single investigator (GMS), who was blinded to HR displayed by ECG and CBF. HR assessments comprised of auscultation using the DS in three different methods: (i) NeoTapLS, (ii) 6 s, and (iii) 10 s. All NeoTapLS, 6 and 10 s HR assessments were assessed from the same starting time point. For NeoTapLS, GMS simultaneously tapped the smartphone screen for each auscultated heartbeat, and the displayed HR was recorded by PAJ. For 6 and 10 s, the number of heartbeats auscultated was verbalized by the assessor (GMS) at 6 and 10 s and recorded by PAJ. GMS was not required to perform arithmetic for determination of HR in bpm; this was determined independently during data analysis. These assessments were repeatedly performed every 30 s in all piglets during asphyxia until asystole. This enabled HR assessment to be performed at various levels of bradycardia, which are representative of different clinical situations (i.e., HR >100, between 60 and 100, or <60 bpm) (11). Markers were placed within the LabChart program to indicate HR assessment times. Post-experiment, the marker was then compared to waveforms from the ECG and CBF to determine HR at the time of assessment using 6, 10 s, and NeoTapLS. HR as determined by CBF was defined as the gold standard (33). Following confirmation of asystole, HR assessments were ceased and interventions were performed according to the study protocol (23).

### Statistical Analysis

All statistical analyses were performed by a statistician who was blinded to the HR assessment approach. Using a single assessor (GMS) eliminated any user bias and error caused by variations between assessors while concurrently allowing for comparison of the same HR at a given point in time. Results from HR assessments are presented as mean (SD). The level of agreement between the measured HR for NeoTapLS, 6 and 10 s intervention groups and CBF HR were assessed using Bland-Altman plots (34). To determine differences at varying HRs during asphyxia, assessments were clustered into subgroups based on CBF HR. *A priori* subgroups were defined as per NRP HR cut-offs (11) HR <60, 60–100, and >100 bpm. Time to assess NeoTapLS, 6 and 10 s are presented as median (IQR). The data was tested for normality and compared using one-way ANOVA with Bonferoni post-test. *P*-values are two-sided and *p* < 0.05 was considered statistically significant. Statistical analyses were performed with Stata (StataCorp, College Station, TX).

## Results

Twenty newborn mixed breed piglets were obtained on the day of the experiment; baseline data and pre-asphyxia parameters are presented in Table 1. The median (IQR) number of assessments per animal was 11 (2–20) observations. A total of 138 HR assessments were performed during asphyxia with 16 observations >100 bpm, 68 observations between 60 and 100 bpm, and 54 observations <60 bpm.

**Table 1:** Baseline and pre-asphyxia parameters.

***n***	**20**
**SEX**	
Female	7
Male	13
Weight (kg)^†^	2.08 (1.8–2.2)
Age (days)^††^	1.85 (1–3)
**BASELINE**	
SpO_2_ (%)	98.8 (97–99)
Heart rate (bpm)	175 (160–204)
MAP (mm Hg)	59.2 (55–71)
CVP (mm Hg)	3.9 (2–5)
pH	7.52 (7.4–7.6)
PaCO_2_ (torr)	36.1 (32.8–40.6)
PaO_2_ (torr)	102.5 (81–130)
BEcf (mmol/L)	4.6 (0–6)
HCO_3_ (mmol/L)	30.09 (24.2–32.81)
**PRE–ASPHYXIA**	
SpO_2_ (%)	30.4 (15–48)
Heart rate (bpm)	256 (187–277)
MAP (mm Hg)	50.8 (36–64)
CVP (mm Hg)	4.6 (2–5)
pH	6.57 (6.5–6.7)
PaCO_2_ (torr)	100.5 (88–113)
PaO_2_ (torr)	14.1 (11–19)
BEcf (mmol/L)	−28.8 (−30 to −26)
HCO_3_ (mmol/L)	9.16 (6.9–11.4)

Data are presented as median (IQR) unless indicated

† mean (SD) or

†† *mean (range)*.

**Figure 1:**
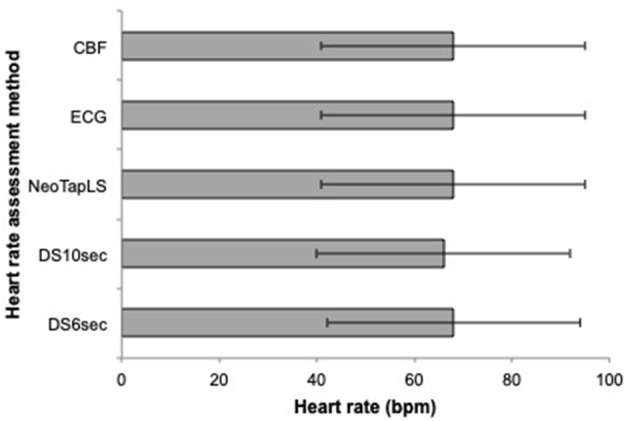
Mean (SD) heart rate during asphyxia assessed using the 6, 10 s, NeoTapLS interventions and standards (ECG and CBF). NeoTapLS, Assessment intervention group using the NeoTapLS smartphone app paired with the digital stethoscope; DS6 s, Assessment intervention group with the 6-s method using a digital stethoscope; DS10 s, Assessment intervention group with the 10-s method using a digital stethoscope; ECG, electrocardiogram; CBF, carotid blood flow.

**Figure 2:**
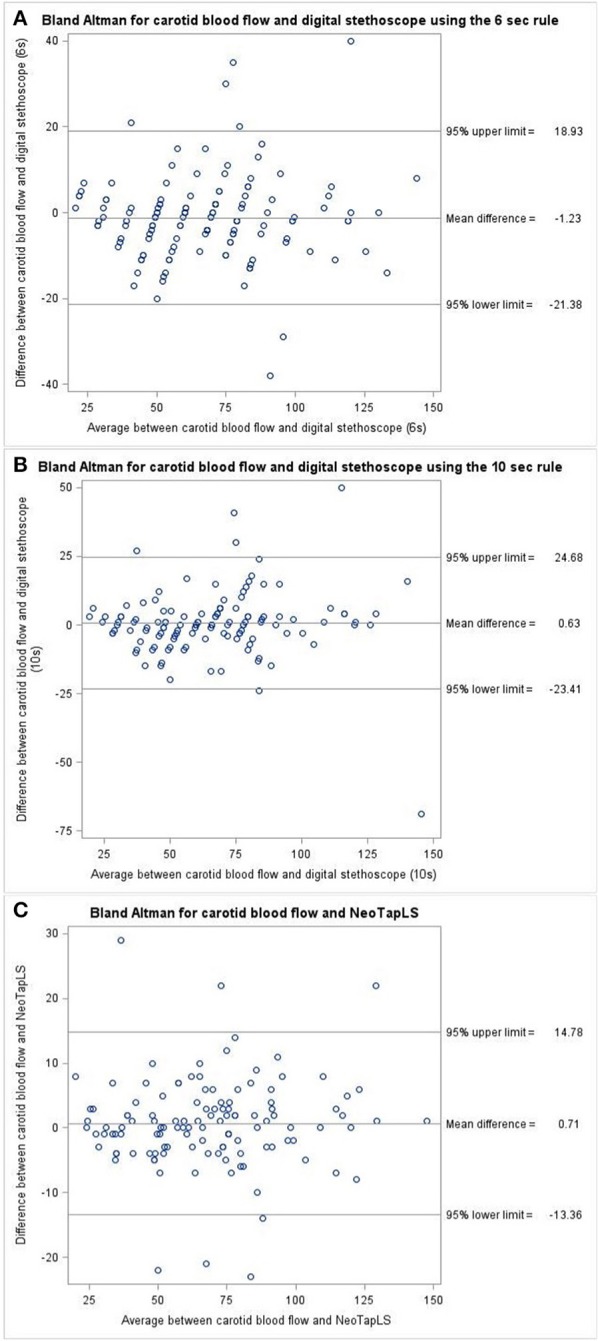
Bland-Altman plot for **(A)** DS6 s vs. CBF, **(B)** DS10 s vs. CBF, and **(C)** NeoTapLS vs. CBF heart rate assessments during asphyxia. CBF, carotid blood flow; DS6 s, digital stethoscope using 6 s method; DS10 s, digital stethoscope using 10 s method; Digital stethoscope paired with NeoTapLS app.

**Figure 3:**
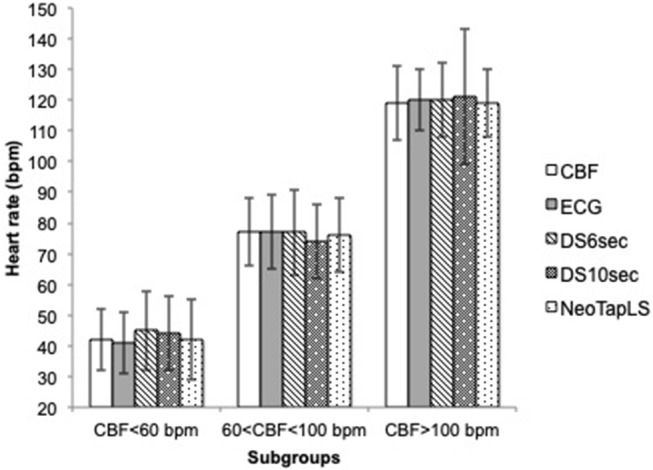
Mean (SD) heart rate during asphyxia assessed by CBF, ECG, DS6 s, DS10 s, and NeoTapLS, according to subgroup 1 (CBF<60 bpm), 2 (60<CBF<100 bpm), and 3 (CBF>100 bpm). DS6 s, DS10 s, NeoTapLS interventions and standards (ECG and CBF). DS6 s, Assessment intervention group with the 6-s method using a digital stethoscope; DS10 s, Assessment intervention group with the 10-s method using a digital stethoscope; NeoTapLS, Assessment intervention group using the NeoTapLS smartphone app paired with the digital stethoscope; ECG, electrocardiogram; CBF, carotid blood flow.

The mean(range) time for asphyxia was 404(72–600) s During asphyxia, the mean (SD) CBF and ECG HR were 68 (27) and 68 (27) bpm, respectively. The HR using NeoTapLS, 6 and 10 s methods were 68 (27), 66 (26), and 68 (26) bpm, respectively (Figure 1). There were no significant differences in the mean(SD) HR measured using CBF, ECG, NeoTapLS, 6 and 10 s methods. However, 95% upper and lower limits of agreement varied for each technique. The Bland-Altman comparisons for CBF HR vs. 6 or 10 s or NeoTapLS are displayed in Figures 2A–C, respectively. Analyses by HR cutoff ranges: <60, 60–100, and >100 bpm are presented in Figure 3.

The median(IQR) time needed to assess HR during asphyxia using the NeoTapLS, 6 and 10 s was 3(2–4), 6(6–7), and 10(10–11) s.

## Discussion

To our knowledge, this is the first study to compare different methods for HR assessment during neonatal asphyxia. Overall, the NeoTapLS had similar accuracy of HR assessment compared to the 6 s method, 10 s methods, ECG, and CBF. Furthermore, subgroup analysis by NRP cut-off values showed similar accuracy of HR assessment. Our study suggests that all evaluated methods have similar accuracy for HR assessments in our asphyxia model. Furthermore, the NeoTapLS method was the fastest technique to assess HR. Our results suggest that the NeoTapLS application might be a useful tool in combination with a stethoscope to assess HR at birth.

In the current study, we observed similar accuracy in HR assessment between the DS, ECG, and CBF. However, there are conflicting results about the accuracy of using the DS to assess a newborn's HR. In the Neonatal Intensive Care Unit and the delivery room, HR assessment using ECG or DS have previously demonstrated low and high accuracy for DS with mean differences of 7.4 and 0.2 bpm, respectively (12, 29). Furthermore, Gaertner et al. reported that the DS only detected HR in 23/37 newborn infants within 30 s (30). In the remaining 14 infants HR could not be assessed due to crying (30). In the current study, all piglets were intubated and sedated/anesthetized and therefore there was no crying/vocalization, which might result in a clearer HR assessment. Moreover, in a real-life resuscitation scenario, crying/vocalization would be unlikely under asphyxia and instead a sign of improved status, where HR assessment and resuscitative interventions might no longer be necessary.

Overall, HR assessment using auscultation has been reported to be inaccurate in 33–75% of cases (20, 35, 36). This could be due to the mental computation required to convert heartbeat counts to HR; however, tap-based applications might reduce these inaccuracies. Furthermore, we speculate time needed to assess HR will take longer in a clinical resuscitation scenario with 10 s as a result of greater cognitive load required for multiplying numbers by 6, in contrast to the 6 s where numbers can easily be multiplied by 10.

Two studies reported that the NeoTapLS application has good accuracy and can be used to quickly assess HR in combination with auscultation during simulated neonatal resuscitation (19, 20). These studies suggest that tap-based mobile applications might have the potential to improve HR assessment in the delivery room. However, studies in the delivery room are lacking. The current study was the first to assess HR during neonatal asphyxia. We observed that the NeoTapLS application paired with the DS had similar accuracy in assessing HR compared to the DS (6- and 10-s rule), ECG, or CBF. Additionally, the NeoTapLS had a shorter median assessment time compared to the 6 and 10-s rule. Since we did not include the time needed for performing mental arithmetic for the 6 and 10-s rule, we speculate the net time for HR assessment will be much longer. Our results suggest that the NeoTapLS application might be useful during neonatal asphyxia to assess HR.

Our Bland-Altman comparisons identified varying upper and low limits of HR assessment using each technique, when compared to the CBF HR. Although all techniques were comparable to the gold standard (CBF HR), NeoTapLS demonstrated the greatest precision for HR assessment as shown by the least difference between 95% upper and lower limits of agreement. This suggests that NeoTapLS offers the higher precision for HR assessment, compared to 6 and 10 s. Despite this, there is persisting variability with NeoTapLS compared to CBF with a wide difference in 95% upper and lower limits of agreement.

We have also identified several limitations for this technology. First, it is only possible to make assessments using both hands, one hand to hold the stethoscope and the other to use NeoTapLS, which means one member of the clinical team must be dedicated for each time HR assessments are performed. Additionally, when using a mobile phone in the delivery room, HR assessments should be the sole purpose and it should be ensured it is thoroughly disinfected before and after use.

## Limitations

Our newborn piglet model is a great strength of this translational study, as this model closely simulates delivery room events, with the gradual onset of severe asphyxia leading to bradycardia and cardiac arrest. However, a few limitations should be considered before implementing these methods in the delivery room. Our asphyxia model uses piglets that were sedated/anesthetized, which is not the case in clinical settings. Our study did not require the assessor to perform mental arithmetic to calculate the HR based off of heartbeat counts when using the 6- and 10-s method; instead the assessor verbally reported the heartbeat count value at 6- and 10-s As the aim for the current study was to compare each HR assessments technique independently, performing the assessment by single assessor (GMS) eliminated user bias and error caused by variations between assessors, while allowing for comparison of the same HR. However, this limits the generalizability of this study to assessors at different skill levels and does not account for time needed to calculate HR following auscultation. Additionally, the current study utilizes the DS together with all methods, which may differ in accuracy compared to these techniques combined with a standard stethoscope. While the DS was used as a standardized tool in this study, it may not readily be available for use in resource-limited regions, as well. Another limitation of our study is that HR <30 bpm was infrequent and thus, difficult to assess accuracy and speed of assessments at extremely low HRs.

## Conclusion

Heart rate assessment with the NeoTapLS application had similar accuracy compared to auscultation with a digital stethoscope with the 6-s method, 10-s method, the electrocardiogram or the carotid blood flow during neonatal asphyxia. The NeoTapLS application had higher precision and a faster time to assess heart rate compared to the 6- or 10-s method with a digital stethoscope. However, clinical trials to evaluate the utility of tap-based applications during neonatal resuscitation are warranted.

## Data Availability Statement

The datasets generated for this study are available on request to the corresponding author.

## Ethics Statement

The animal study was reviewed and approved by Approval of the Animal Care and Use Committee (Health Sciences), University of Alberta (AUP00002151), presented according to the ARRIVE guidelines, and registered at preclincialtrials.eu (PCTE0000155).

## Author Contributions

GS, PY-C, and MO'R: conception. GS, NM, T-FL, MO'R, P-YC, and PJ: data acquisition, data analysis, interpreting of results, drafting of the manuscript, critical revision of the manuscript, and final approval of the manuscript.

### Conflict of Interest

The authors declare that the research was conducted in the absence of any commercial or financial relationships that could be construed as a potential conflict of interest.

## Abbreviations

HR: Heart rate
bpm: Beats per minute
ECG: Electrocardiography
PO: Pulse oximetry
SpO_2_: Oxygen saturation
NRP: Neonatal Resuscitation Program
ERC: European Resuscitation Council
CBF: Carotid blood flow
NeoTapLS: NeoTapLifeSupport app
6sec: 6-sec method
10sec: 10-sec method
SD: Standard deviation
IQR: Interquartile range.
